# A post pandemic roadmap toward remote assessment for neuromuscular disorders: limitations and opportunities

**DOI:** 10.1186/s13023-021-02165-w

**Published:** 2022-01-04

**Authors:** Jacqueline Montes, Katy J. Eichinger, Amy Pasternak, Cara Yochai, Kristin J. Krosschell

**Affiliations:** 1 Department of Rehabilitation and Regenerative Medicine, Columbia University Irving Medical Center, 617 West 168th Street, Room 347, New York, NY 10002 USA; 2 Department of Neurology, University of Rochester, Rochester, NY USA; 3 Departments of Physical Therapy and Occupational Therapy Services and Neurology, Boston Children’s Hospital, Boston, MA USA; 4 Department of Neurology, Columbia University Irving Medical Center, New York City, NY USA; 5 Department of Physical Therapy and Human Movement Sciences and Department of Pediatrics, Northwestern University Feinberg School of Medicine, Chicago, IL USA

**Keywords:** Neuromuscular disorders, Outcome measure, Telehealth, Remote assessment, Clinical trials, Clinical outcome assessments (COA)

## Abstract

Recent advances in technology and expanding therapeutic opportunities in neuromuscular disorders has resulted in greater interest in and development of remote assessments. Over the past year, the rapid and abrupt COVID-19 shutdowns and stay-at-home orders imposed challenges to routine clinical management and clinical trials. As in-person services were severely limited, clinicians turned to remote assessments through telehealth to allow for continued care. Typically, disease-specific clinical outcome assessments (COAs) for neuromuscular disorders (NMD) are developed over many years through rigorous and iterative processes to fully understand their psychometric properties. While efforts were underway towards developing remote assessments for NMD before the pandemic, few if any were fully developed or validated. These included assessments of strength, respiratory function and patient-reported outcomes, as well as wearable technology and other devices to quantify physical activity and function. Without many choices, clinicians modified COAs for a virtual environment recognizing it was not yet known how they compared to standard in-person administration. Despite being able to quickly adapt to the demands of the COVID-19 pandemic, these experiences with remote assessments uncovered limitations and opportunities. It became clear that existing COAs required modifications for use in a virtual environment limiting the interpretation of the information gathered. Still, the opportunity for real-world evaluation and reduced patient burden were clear benefits to remote assessment and may provide a more robust understanding and characterization of disease impact in NMD. Hence, we propose a roadmap navigating an informed post-pandemic path toward development and implementation of safe and successful use of remote assessments for patients with NMD.

## Introduction

Advances in technology over the past two decades have led clinicians to reconsider how clinical care is administered and research is conducted. The ability to collect information on patients or research participants remotely offers several benefits particularly in rare conditions, such as neuromuscular disorders (NMD). Travel to tertiary care centers for expert care or extended clinical trial visits are often challenging because of accessibility, need for assistance, and prolonged time away from home or work. Incorporating remote assessments in patient management and study design allows for data to be collected more frequently and in a person’s natural or home environment, while reducing the burden of the number of in-person visits.

With the expansion of therapeutic opportunities in NMD, there is a common need to describe the evolving natural history and responses to treatment. Outcome measures that quantify impairments and functional abilities are paramount to understanding the natural history and evaluating treatments. Measures of strength and function have been the cornerstone for assessments in NMD as the prevailing symptoms are often weakness, altered motor development or impaired functional abilities. The psychometric properties of the most commonly used clinical outcome assessments (COAs) for NMD are well-established through iterative processes and rigorous examination. These established COAs may inform the development of remote assessments.

The COVID-19 pandemic catapulted the utilization of telehealth and remote assessment, with limited experience amongst clinicians [1]. Multidisciplinary teams scrambled to find ways to monitor disease progression, evaluate response to treatment and assess the needs of patients as traditional COAs were no longer possible. At the time, a limited number of remote assessments in the early stages of development were available, several requiring equipment or devices, and none were well established. To maintain continuity of care and adapt to a remote environment, interim strategies for assessment using COAs, despite lack of validation for remote settings, were implemented during NMD clinic telehealth visits. As in-person care resumes and we move back to the clinic, efforts to further develop and validate remote assessments, informed by this pandemic experience, are warranted. The benefits of real-world evaluation and reduced patient burden using remote assessment is clear. A systematic and thoughtful approach to developing remote assessment in NMD is critical.

### Pre-pandemic approach to remote assessment

Clinically, providers have been interested in developing methods to assess individuals with neuromuscular conditions via telehealth for more than a decade. Several modes of providing telehealth are utilized but the terminology used to describe it varies (Table 1). Telehealth, encompasses all aspects of health-related activities through internet or other electronic means (not in person) including clinical, administrative and educational services [2, 3]. Increasing access to healthcare is one of the reasons that telehealth was first used as early as 2008 [4]. Improved access to providers for individuals living in rural areas was an early benefit. In addition, decreasing the time spent traveling to the clinic, expenses accumulated while traveling and time out of work could be seen as a benefit for families, especially for those who live a great distance from their providers [5]. While available, telehealth was rarely implemented by most clinicians in tertiary and or urban settings.

**Table 1:** Remote assessment terminology

Term	Definition
**Services**	
e-health	The use of information and communication technologies for health [6]
Remote assessment	Includes any type of remote monitoring (e.g., wearables, digital technology, clinician-administered assessments or outcome measures, patient reported outcomes) using internet, phone, or electronic means (not in person) [2]
Telehealth	The use of electronic information and telecommunication technologies to remotely provide health care information and services. Includes all aspects of health-related activities through internet or other electronic means (not in person) including clinical, administrative and educational services [2, 3]
Telemedicine	Refers specifically to delivery of clinical health-related services via internet or electronic means (not in person) [7]
Telerehabilitation	Refers specifically to clinical rehabilitation services with the focus of evaluation, diagnosis, and treatment [8]
**Types of Remote Assessment**	
Bio-telemetry	The remote detection and measurement of a human or animal function, activity, or condition (such as heart rate or body temperature)
Clinical Outcome Assessment. (COA)	A measure that describes or reflects how a patient feels functions or survives [9] *Clinician Administered Assessment of Performance:* A measurement based on standardized tasks actively undertaken by a patient according to a set of instructions. May be administered by an appropriately trained individual or completed by the patient independently *Patient Reported Outcome Measure (PRO):* A measurement based on a report that comes directly from the patient about the status of a patient’s health condition without amendment or interpretation of the patient’s response by a clinician or anyone else
Digital Biomarkers	Objective, quantifiable, physiological, and behavioral data that are collected and measured by means of digital devices such as portables, wearables, implantables, or digestibles. The data collected are usually used to explain, influence, and or predict health related outcomes [10]
Digital Health Technology	Uses software, sensors, connectivity, and other computing platforms for health care and related uses. Digital health more broadly includes wearables, telehealth, and telemedicine [9]
Remote Patient Monitoring	Type of ambulatory healthcare where patients use mobile medical devices to perform a routine test and transmit the test data to a healthcare professional [11]
Wearables	Sensors or applications that can collect health related data in-person or remotely [12]

Recent efforts, initiated prior to the pandemic, towards developing remote assessments in NMD have focused on strength, respiratory function and patient-reported outcomes [13–15]. Activity monitoring with commercial or research grade devices have been used to evaluate gait and physical activity [13, 16–20]. However, few of these tools have been validated and there is little information on feasibility or barriers to use. Digital health technology such as customized phone apps developed to assess upper limb function and mobility are in early exploratory phases [13, 21]. Validation of these technologies and refinement of the study methodology is still needed to reduce variability and enable wide adoption for clinical trial purposes [22].

There is inconclusive and conflicting evidence supporting the reliability of remote assessment. Several studies in disorders other than NMD, demonstrate adequate inter-rater agreement between telehealth and face-to-face clinical assessments [23–26]. In contrast, mobility parameters obtained in different settings with participants with neurologic disorders, including Parkinson’s disease, differed significantly, with differences much larger than effects measured after an actual intervention [27]. To our knowledge, there are no published studies evaluating established in-person COAs administered remotely in individuals with NMD.

### Transformation of remote assessment during the pandemic

The rapid and abrupt transition due to COVID-19 shutdowns and stay-at-home orders imposed challenges to clinical management and on-going clinical trials for the NMD community. Existing standards of care supported the assumption that in-person assessments remained critical for differential diagnosis, assessment of motor function, and reimbursement from third-party payers for the provision of therapeutic interventions. However, local travel and hospital mandates and personal risk led to an upsurge in telehealth. As in-person services were severely limited, telehealth was rapidly implemented, despite a lack of prior experience at most institutions, to allow for continued care. Providers immediately adjusted the delivery of care, adapted to new regulatory constraints, and adopted new technology and terminology. Because of the urgency of the moment, disease specific standardized outcome measures were performed remotely with ad hoc adaptations in attempt to get the best possible representation of a patient’s status. The usual structured in-clinic procedures to administering assessments were superseded by desperate need for some measure of disease status.

Initial strategies for telehealth and remote assessments involving individuals with NMD focused on establishing a safe and feasible environment with adequate technological support. In this environment, challenges of adequate space, digital access and literacy, and equipment for testing were revealed [28, 29]. Connectivity via broadband was limited for some, particularly for underserved populations, introducing unwanted bias [30]. Videoconferencing was challenging for those who were less technologically savvy or those who relied on eye-gaze, text to voice apps or caregiver support to connect. These issues impacted ability to complete and interpret most physical exam items and COAs that were attempted in the remote setting.

Younger as well as more impaired individuals with limited motor function relied on caregivers for accessing and participating in virtual visits. Higher functioning individuals had fewer physical restrictions but required more guidance during challenging functional assessments to ensure safety. Initial successful telehealth visits included subjective reports provided by the patient and caregivers and a brief physical exam, including active range of motion and functional observation. As the pandemic and in-person visit restrictions continued, the need to better assess disease progression or response to interventions increased. This led clinicians to informally explore the potential feasibility of the administration of disease-specific standardized functional assessments (COAs), as well as impairment-based assessments that traditionally require an in-person hands-on approach. Attempts at timed function tests like "floor to stand” and 10-m walk/run revealed lag time across internet servers. Completion of functional COAs were often limited by an individual’s ability, environmental constraints, or suboptimal camera views resulting in missing items and partial scores. Therefore, extrapolating test scores obtained during telehealth did not always translate to the scores obtained during in-clinic assessments.

Despite the challenges imposed by the pandemic, it is evident that a shift towards formally incorporating telehealth and remote assessment into NMD patient care and clinical trials is imminent and necessary. A new battery of COAs adapted for the virtual environment needs to be developed and/or validated. There are clear benefits to telehealth and remote assessments. The ability to observe an individual perform functional mobility tasks and self-care (ie. climbing stairs, transferring, getting dressed) in their natural environment is meaningful and invaluable.

### Proposed roadmap for post-pandemic remote assessment

Despite being able to quickly adapt to the demands of the COVID-19 pandemic, telehealth experiences have uncovered limitations in and opportunities for remote assessments. Clinicians, researchers, and other stakeholders committed to exploring the boundaries of remote assessments of individuals with neuromuscular conditions should understand the foundational groundwork necessary to develop and validate assessments with the same rigor that has been used to develop currently accepted COAs. Understanding the feasibility of remote assessments to evaluate strength and function is fundamental to this work. Feasibility varies by type of assessments. Surveys and patient reported outcomes have higher feasibility and will require less time to establish efficacy in remote settings, whereas clinician administered motor function remote assessment and digital monitoring will require additional work and time to establish reliability and validity. Disease specific outcome measures are optimal in assessment of NMD patients particularly considering the therapeutic landscape and should be prioritized in the development of remote assessment.

**Fig. 1:**
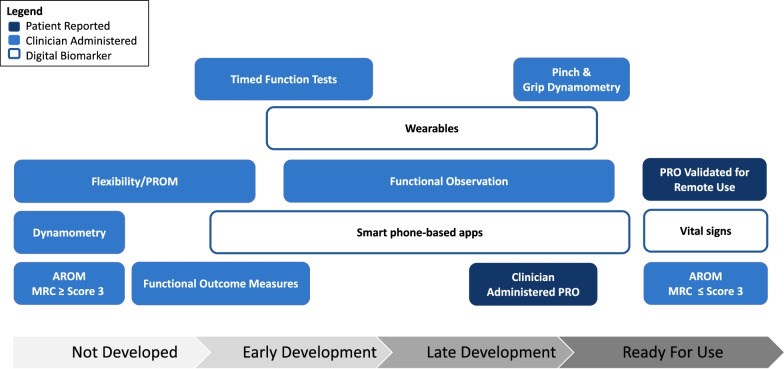
Types of remote assessment: development and readiness for use. Outcomes are presented from left to right based on stage of development for remote use (Not Developed, Early Development, Late Development, and Ready for Use) and type of assessment (Patient-Reported = dark blue, Clinician Administered = light blue, Digital Biomarker = white). AROM = active range of motion; PROM = passive range of motion; MRC = medical research council; PRO = patient-reported outcome measure)

Expert opinion and consensus-based recommendations are important contributions to identifying feasibility of remote assessments for different populations. COAs for use in the remote environment are in various stages of development (Fig. 1). Ideally, COAs identified as ready-to-use will have clearly established relationships with traditional in-clinic assessments. Identification of safe and practical assessments, appropriate for remote administration, is also necessary. In the meantime, patient-reported outcome measures whether adapted for remote collection or clinician administered can be implemented without additional adaptation or with very minimal development. Many of these measures have been developed as computer adaptive instruments or have online versions ready for use [31, 32]. Patients and caregivers can be instructed on active and passive range of motion activities to be assessed by the clinician remotely. Digital technology, smart-phone based apps and wearable technology is currently available with some evidence for feasibility, and performance in NMD can be incorporated. Selecting technology for remote assessments that features “usability” and easy set-up to reduce burden on the user needs to be considered. In some cases patients and caregivers might be able to leverage their own devices and resources (iPhone, iPad), however, researchers (and sponsors) will need to provide adequate support for remote visits in clinical trials when families experience issues with any technology that is implemented.

**Fig. 2:**
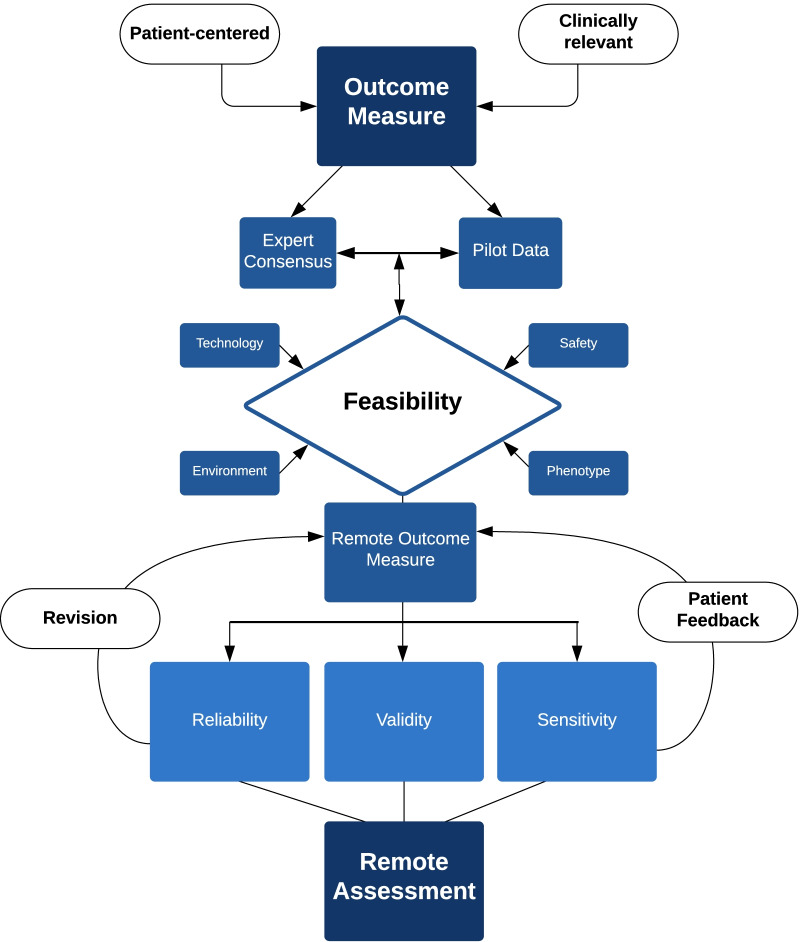
Roadmap for the development of remote assessment. This figure depicts the interactive and operational processes to develop a remote assessment. The road map outlines a framework for choosing an outcome measure, exploring feasibility, and then determining its validity, reliability and sensitivity to change in a remote settting

Remote assessments, whether supervised or derived from digital health technology, require established validity, reliability, and sensitivity to change. Existing measures should not simply be retrofit to the virtual environment presuming similar attributes established during in-person visits. However, the path for each remote assessment will depend on its stage of development (Fig. 1). Moreover, the purpose of the remote assessment should be well-defined a priori. For example, some assessments may serve as interim assessments to COAs performed in clinic and others may provide complementary evaluation of real-world performance and experiences. It is critical to examine the reliability, validity, and sensitivity to change with treatment and over time of these remote assessments in an iterative process (Fig. 2). Utilizing a methodological approach that meets established guidelines (https://www.fda.gov/about-fda/center-drug-evaluation-and-research-cder/division-clinical-outcome-assessment-dcoa) and incorporating regulatory and patient feedback is essential. Some remote assessments have undergone feasibility and psychometric testing, permitting an abbreviated timeline entering the roadmap at later stages. Gathering expert consensus and stakeholder feedback can be facilitated through virtual interactions, perhaps under-utilized prior to the pandemic, which would further expedite the process. Regardless, until this iterative process occurs, the ability to compare in-person data to remote data will be difficult, and the capacity to interpret change across both remote and in-person settings will remain limited.

Careful consideration to develop and deliver valid and reliable COAs for remote use is critical. As a result, remote assessments that are psychometrically sound, meaningful and sensitive to change will be available for use in NMD. Incorporating the lessons learned during the pandemic and in an attempt to circumvent failure and promote success, we propose recommendations for the development and delivery of remote assessments for individuals with NMD. Moving forward, remote assessment can expand upon an in-person assessment and provide a more comprehensive evaluation of the patient experience. Clinical trials could be adapted to include remote assessments to reduce burden on patients and clinical sites without gaps in monitoring. Additionally, remote assessment could foster access to participants outside of a clinical site’s geographical region. This would create opportunities to reach patients with limited clinical services, expertise and resources.

Successful translation of COAs to the virtual environment is dependent on the type of assessment, as well as the patient’s age, disease, phenotype, and environment. The roadmap (Fig. 2) to timely and effective adoption of remote outcome measures for use in NMDs should include: (1) identifying appropriate patient-centered and clinically relevant measures, (2) establishing recommendations to assess readiness for use of promising remote assessments, (3) determining the psychometric properties of the remote assessments, (4) comparing in-person data to remote data to understand change across both remote and in-person settings for clinician administered and patient reported outcomes (5) integrating the remote assessments into study design and clinical practice, (6) encouraging engagement of and feedback from user groups (clinicians and patients), and (7) insuring access to the internet, technology and equipment necessary to complete the remote assessments. This suggested road map will lead to improved remote assessments of individuals with NMD, address regulatory requirements for use in clinical trials, and enhance health care outcomes. Remote assessments should be considered exploratory until administration standards and psychometric properties are well-defined. In the meantime, clinicians and researchers should rely on well-established in-person assessments to evaluate disease status in patient management and clinical trials.

## Conclusions

Advances in technology initiated interest in remote assessments of individuals with NMD both for clinical care and research endeavors. The COVID-19 pandemic created an environment that resulted in rapid transformation of how clinical care was delivered and research was conducted leading to an increased understanding of the benefits that remote assessment can bring to the care of those with NMDs. Across the lifespan of individuals with NMD, there are opportunities to capture objective and meaningful information regarding a person’s strength, function, and daily performance using remote assessments. The addition of real-world assessments may provide a more robust understanding and characterization of disease impact in NMD. The combined use of in-person and remote assessments may provide a more holistic view of the clinical manifestations and patient experience. Clinical trials could be adapted to include remote assessment to expand recruitment, facilitate retention, and reduce patient burden. Clinicians and researchers need to proceed with caution, in order to ensure safe, accurate and meaningful use of this modality. Risks of utilizing COAs established for in-person use through telehealth prematurely may lead to inaccurate characterization of clinical status and change. This roadmap provides a starting point for a measure’s readiness for use and a way to determine what measurement attributes need further work prior to use in a telehealth setting. Steps to guide the path to clinically valid and reliable remote COAs should mimic previous approaches utilized to achieve already well- established in person COAs.

## Data Availability

Not applicable.

## Abbreviations

COA: Clinical outcome assessment
NMD: Neuromuscular disorder
